# Genital bar electrode for dorsal genital nerve stimulation: feasibility and safety of a novel approach for managing bowel dysfunction and overactive bladder

**DOI:** 10.1007/s10151-026-03386-6

**Published:** 2026-07-09

**Authors:** L. S. Grau, P. Christensen, M. El Issaoui, N. Klarskov, M. B. Ellebæk, N. Qvist, C. Graugaard-Jensen, M. Glavind-Kristensen, J. Duelund-Jakobsen

**Affiliations:** 1 Department of Surgery, Pelvic Floor Unit, Aarhus University Hospital, Aarhus, Denmark; 2 Department of Clinical Medicine, Aarhus University, Aarhus, Denmark; 3 Obstetrics and Gynaecology, Herlev University Hospital, Herlev, Denmark; 4 Department of Clinical Medicine, University of Copenhagen, Copenhagen, Denmark; 5 Research Unit for Surgery, Odense University Hospital, Odense, Denmark; 6 Department of Clinical Research, University of Southern Denmark, Odense, Denmark; 7 Department of Urology, Pelvic Floor Unit, Aarhus University Hospital, Aarhus, Denmark; 8 Department of Gynaecology and Obstetrics, Pelvic Floor Unit, Aarhus University Hospital, Aarhus, Denmark; 9 University of Southern Denmark, Odense, Denmark

**Keywords:** Bowel dysfunction, Dorsal genital nerve stimulation, Faecal incontinence, Faecal urgency, Overactive bladder, Piercing

## Abstract

**Background:**

Electrical stimulation of the dorsal genital nerve (DGN) using a patch electrode is effective in treating bowel dysfunction (BD) with faecal urgency and/or incontinence and overactive bladder (OAB). Keeping the patch electrode in place during stimulation may be challenging. This study evaluated the feasibility and safety of inserting a novel genital bar electrode for DGN stimulation.

**Method:**

A vertical clitoral hood piercing with a specially designed genital bar electrode was inserted under aseptic conditions with topical anaesthesia in women who had undergone DGN stimulation via a patch electrode, and reported subjective symptom improvement. After a 4-week healing period, stimulation with the bar electrode was initiated, followed by completion of a similar symptom diary to that with the patch electrode.

**Results:**

All 14 patients included in the study completed it. No procedure-related complications occurred. During the healing period, 10 patients experienced minor transient adverse events: mild bleeding or pain (*n* = 7 each), swelling (*n* = 4), redness (*n* = 3), and isolated numbness, irritation, or itching (*n* = 1 each). Nine patients experienced stimulation-related adverse events, mainly local pain or discomfort. Granulation tissue developed in one case. Urgency and incontinence episodes were reduced by a median of 70% and 59%, respectively, during genital bar electrode stimulation, with symptom responses similar to those observed with patch electrode stimulation.

**Conclusion:**

Inserting a genital bar electrode is feasible and safe. Preliminary results indicate a symptom response comparable to patch electrode stimulation in the majority.

**Supplementary Information:**

The online version contains supplementary material available at https://doi.org/10.1007/s10151-026-03386-6.

## Introduction

Bowel dysfunction (BD) with faecal urgency (FU) and/or faecal incontinence (FI) and overactive bladder (OAB) are prevalent and debilitating conditions that considerably affect quality of life [1–3]. FU prevalence in the general population varies from 3.3% to 14.4% among healthy individuals [4, 5] and up to 65% among patients with BD [6]. FI affects approximately 7% of the adult population [7]. FU is a sudden, urgent need to defecate that cannot be postponed and may be accompanied by fear of incontinence [8], while FI refers to the inability to control bowel movements, which can lead to unintentional stool passage (Rome IV criteria) [8]. The estimated prevalence of OAB in Western countries ranges from 16.8% to 43%, and it increases with age [9, 10]. OAB is characterised by urinary urgency, with or without incontinence, often with associated frequency and nocturia [11].

Conservative treatment is the first-line approach for both FU/FI [12] and OAB [13]. FU has no established therapy [14], and FI is managed with pelvic floor exercises, dietary modifications, constipating agents, and second-line irrigation [12]. OAB treatment progresses from behavioural strategies often combined with pelvic floor exercises to second-line treatments, such as vaginal oestrogen for postmenopausal women, antimuscarinics or β3-adrenoceptor agonists, with onabotulinumtoxinA reserved for third-line therapy [13]. In many patients, achieving adequate symptom control remains challenging despite optimal conservative treatment [15–17]. Neuromodulation therapies, including percutaneous tibial nerve stimulation (PTNS) and sacral neuromodulation (SNM), provide treatment options in such cases. PTNS has shown promising results in the management of BD [18]; however, a randomised controlled trial has not consistently demonstrated a significant treatment effect [19]. PTNS appears effective in reducing urgency and may improve incontinence symptoms in some patients with OAB; however, treatment response remains variable and not all patients achieve satisfactory symptom control [20]. SNM has proven to be effective for both BD [21] and OAB [22]. However, SNM is a costly and invasive procedure with a risk of complications that may necessitate further surgical interventions [23].

Dorsal genital nerve (DGN) stimulation has emerged as a promising neuromodulation alternative for the treatment of both BD [24, 25] and OAB [26]. Traditionally, DGN stimulation is delivered using a genital patch electrode; however, this type of electrode has practical difficulties, including detachment, replacement, and difficulty with proper positioning [27]. To address these difficulties, a novel genital bar electrode, resembling a genital piercing accessory, has been developed for vertical clitoral hood piercing.

The primary aim of this study was to describe in detail the feasibility and safety of insertion and initial stimulation of the genital bar electrode for DGN stimulation. The secondary aim was to explore preliminary symptom responses of genital bar electrode stimulation and to assess whether its effects resemble those observed with patch electrode stimulation.

## Materials and Methods

### Ethics

Patients received both written and oral information about the study and provided written informed consent. The Danish Research Ethics Committees (CIV-23-07-043535) and the Danish National Medical Research Ethics Committee (VMK: 2311405) approved the study. The study was conducted in accordance with the Declaration of Helsinki [28]. The clinical investigation protocol was registered at ClinicalTrials.gov NCT06091566. Data were stored and processed in accordance with the General Data Protection Regulation (GDPR) [29]. In the event of any unexpected adverse events related to the study, patients were covered by national and product liability insurance.

Patients were recruited at the Pelvic Floor Clinic, Department of Surgery, Aarhus University Hospital (AUH); the Obstetrics and Gynaecology Department, Herlev University Hospital (HH); and the Department of Surgery, Odense University Hospital (OUH). Recruitment occurred between January 2024 and February 2025 in accordance with the inclusion and exclusion criteria (Table 1).

**Table 1:** Inclusion and exclusion criteria

Inclusion	Exclusion
Women ≥ 18 years	Medically unstable condition
Clitoral hood depth of ≥ 10 mm (confirmed by cotton-swap test)	Active infection in the genital area
*Bowel dysfunction indication* At least one of the following symptoms Faecal incontinence (urge/passive) ≥ 1 episode/week Faecal urgency ≥ 3 episodes/week together with a St. Mark’s Incontinence Score ≥ 9	Implanted pacemaker Implanted drug pump Other active medical devices that use electrical energy or other source of power for function
*Overactive bladder indication* Urinary urgency usually with one or more of the following Urgency urinary incontinence ≥ 1 episode/3 days Urinary frequency ≥ 8 voids/day without polyuria Nocturia ≥ 2 voids/night without nocturnal polyuria	OnabotulinumtoxinA treatment in the pelvic region within the past 3 months Use of antimuscarinics or β3-adrenoceptor agonists within the past 2 weeks Initiation of oestrogen therapy within the past 3 months or intention to discontinue during the study
Communicate, provide feedback, understand and follow instructions in Danish	Participating in another investigational study or drug/medical device trial within 4 weeks prior to enrolment
Failed conservative treatment	Neuropathy
Negative pregnancy test (human chorionic gonadotropin urine test)	History of, or ongoing cancer treatment in the pelvic region
Effective contraception*	Addictive behaviour**
	Contraindications for genital bar electrode insertion Lichen sclerosus Anatomical issue that could prevent proper placement

*Contraception considered effective: intrauterine device, hormonal contraceptives such as birth control pills, implants, vaginal rings, or contraceptive injection

**Addictive behaviour is defined as the abuse of alcohol, cannabis, opioids, or other intoxicating drugs

Patients with BD completed a 14-day baseline bowel symptom diary, and those with OAB completed a 3-day baseline bladder symptom diary (Table 2). Urgency episodes were graded according to patient-reported severity using a grading system adapted from the Patient Perception of Intensity of Urgency Scale (PPIUS) [30] (Table 2). Episodes scored as *grade 3 and 4* represented severe urgency and urgency incontinence and were pre-specified as the primary urgency outcome.

**Table 2:** Symptom diary outcomes for bowel dysfunction and overactive bladder. Urgency episodes were graded according to patient-reported severity

Bowel dysfunction		Overactive bladder	
Diary	Defecation: Made it to the toilet Did not reach the toilet	Diary	Urination: Made it to the toilet Did not reach the toilet
	Urgency level: 1. None: Went to the toilet for other reasons 2. Mild: Able to postpone defecation for a long time without fearing incontinence 3. Moderate: Able to postpone defecation for a short time without fearing incontinence 4. Strong: Unable to postpone defecation in risk of incontinence 5. Urge incontinence		Urgency level: 1. None: Went to the toilet for other reasons 2. Mild: Able to postpone urination for a long time without fearing incontinence 3. Moderate: Able to postpone urination for a short time without fearing incontinence 4. Strong: Unable to postpone urination in risk of incontinence 5. Urge incontinence
	Incontinence: Urge incontinence Passive incontinence		Leaked urine: None A bit Moderate A lot
			Stress urinary incontinence: Yes No

Following baseline symptom diary assessment, patients entered a 4-week stimulation screening period using patch electrodes and the external UCon neurostimulator [27]. Stimulation parameters consisted of charge-balanced square pulses (200 μs, 20 Hz, 0–25 mA), with intensity individually adjusted below the pain threshold. Home-based DGN stimulation was performed either for 30 min daily or on demand (60 s stimulation upon urgency experienced throughout the day) [27]. The patch electrode was applied only during stimulation sessions. After completion, a similar symptom diary to that at baseline was completed. Patients who reported a Patient Global Impression of Improvement (PGI-I) score ≤ 3 (1 = significantly better; 7 = significantly worse) [31] were eligible to proceed with the insertion of the genital bar electrode.

Following genital bar electrode insertion, a minimum of 4 weeks of healing with weekly follow-ups was required before electrical stimulation was initiated. Then, a 4-week stimulation period was conducted using the inserted genital bar electrode, followed by completion of a final symptom diary, as at baseline. Stimulation parameters and treatment instructions were identical to those used during the screening period with the patch electrode and using the same external UCon neurostimulator [27]. The bar electrode remained in situ throughout the study period; however, it was connected to the external stimulator only during stimulation sessions.

Safety was evaluated using patient-reported adverse or device-related events during insertion, healing, and stimulation. Symptom response outcomes were derived from symptom diary measures collected at baseline and at the end of each stimulation period.

### Insertion procedure development

A procedure protocol was developed before inserting the genital bar electrode. During protocol development, the study team conducted online consultation meetings with an experienced, internationally recognised piercer [32], and practical training on the insertion technique was performed using chicken skin as a model. Each centre designated a single trained clinician to perform all insertions.

#### Insertion procedure

##### Procedure

**Fig. 1:**
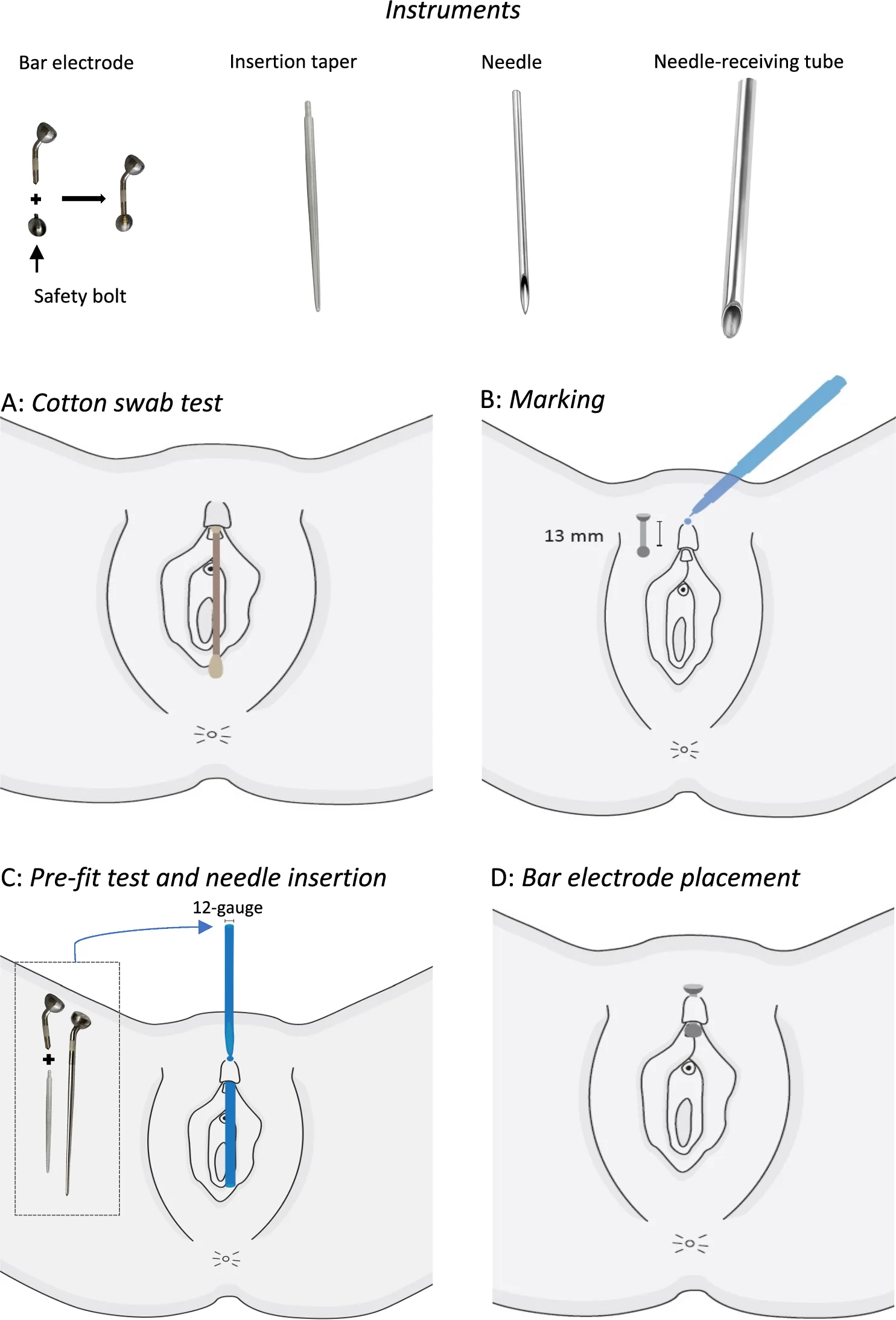
Insertion procedure guide. **A** A cotton swab is placed beneath the clitoral hood to confirm a minimum of 10 mm depth. **B** The insertion site is marked at the superior edge of the clitoral hood, 13 mm from the posterior edge. **C** A pre-fit test is performed using the bar electrode and insertion taper. The needle perforates the skin and enters the receiving tube, after which the genital bar electrode is advanced using the insertion taper. **D** The insertion taper, needle and receiving tube are removed, and the safety bolt is positioned on the genital bar electrode and secured

Insertion of the genital bar electrode took place in an outpatient clinic under aseptic conditions with topical anaesthesia (Fig. 1). No prophylactic antibiotics were administered before or after the procedure. Patients were prescribed oral paracetamol 1000 mg and ibuprofen 400 mg 2 h before the procedure. With the patient in the lithotomy position and the legs supported in stirrups, the cotton swab test was repeated (Fig. 1A) to confirm eligibility. The genital area, including the region beneath the clitoral hood, was thoroughly disinfected with chlorhexidine 0.05%, applied twice. Topical anaesthesia (Xylocaine 10 mg spray) was applied afterwards. After 15 min, a marking 13 mm from the edge of the clitoral hood towards the apex was performed with a sterile pen (Fig. 1B), facilitating correct alignment and preventing the safety bolt from being positioned beneath the clitoral hood. To provide adequate access for the procedure, the clitoral preputium was stretched by pulling the edge of the clitoral hood. A pre-fit test (Fig. 1C) with the insertion taper and the genital bar electrode was performed to ensure correct genital bar electrode placement. An 8-gauge needle receiving tube was placed beneath the clitoral hood (Fig. 1C), ensuring the pen mark was aligned with the entrance of the tube. The 12-gauge needle, lubricated with sterile gel, was advanced into the receiving tube, perforating the tissue at the marked point (Fig. 1C). The genital bar electrode was tunnelled through the tissue using the lubricated insertion taper (Fig. 1C). After the genital bar electrode was successfully positioned, the needle, insertion taper, and the tube were carefully withdrawn. Finally, the safety bolt was securely fastened to the base of the genital bar electrode (Fig. 1D).

##### Instruments

Genital bar electrode (diameter 2.0 mm and 25 mm length, weight 1.3 g, PEEK (Vestakeep i4R) plastic, Ti6Al4V extra low interstitial titanium, a hermetically encapsulated NdFeB magnet), safety bolt, 12-gauge pin-coupled insertion taper, 12-gauge body piercing needle, 8-gauge needle receiving tube (Fig. 1).

##### Post-procedure

Patients received detailed oral and written instructions for postoperative care (Appendix A). This included advice to clean the area with a 0.9% saline solution three to five times daily. Proper hand hygiene was emphasised, encouraging patients to thoroughly wash their hands before touching the genital bar electrode or genital area during the initial weeks. Patients were instructed to avoid using local care products, bathtubs, and swimming pools for 2 weeks, and to refrain from physical activities such as cycling or horseback riding during the healing period. Sexual activity was permitted but should be stopped if pain or tenderness occurred. Minor bleeding was expected and could be managed with sanitary pads. Tight cotton underwear was recommended to stabilise the genital bar electrode and prevent local trauma. Patients were instructed to seek immediate medical attention if signs of severe bleeding or infection occurred.

### Statical analysis

Patients served as their own controls. Data were examined for normality before analysis. Change in symptoms was expressed as an individual percentage delta value, calculated as [(baseline − intervention)/baseline] × 100, with positive values indicating improvement and negative values indicating worsening. Median percentage delta changes with interquartile ranges (IQR; i.e. 25–75, Q1–Q3) were calculated separately for bar electrode and patch electrode stimulation for both urgency and incontinence. Differences between interventions were assessed using the Wilcoxon signed-rank test, with* p* values ≤ 0.05 considered statistically significant. A sensitivity analysis, including patients with OAB and patients with BD, evaluated the feasibility of pooling the data. Patient Global Impression of Improvement (PGI-I) was analysed descriptively. Analyses were performed using Stata version 18.5 (Stata Corporation, 4905 Lakeway Drive, TX, USA).

## Results

**Fig. 2:**
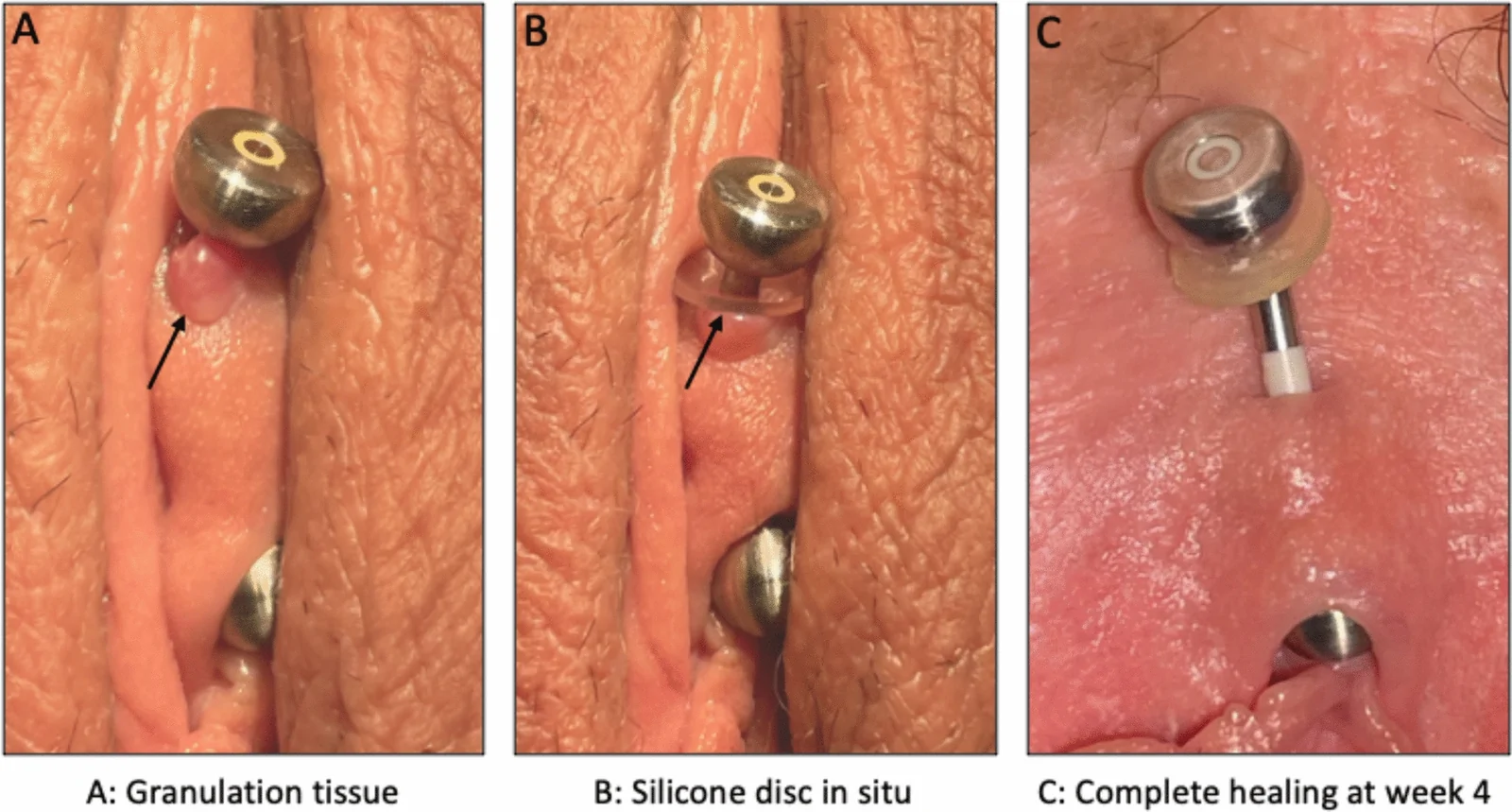
**A** Granulation tissue treated with a silicone disc for compression (**B**), with complete healing after 4 weeks (**C**). (Clinical photo with permission from patient)

Twenty-eight patients were eligible; 14 were included and underwent insertion of the genital bar electrode (Fig. 2). The median age was 52 years (IQR 36–61). Symptom duration was a median of 18 years (IQR 7–25). Ten patients had a history of vaginal delivery. Three were diagnosed with BD, and 11 with OAB (Fig. 2). The underlying aetiology in all patients with BD was idiopathic. For patients with OAB, the aetiology was idiopathic in nine and neurological in two.

### Safety outcomes for insertion procedure and 4-week healing period with genital bar electrode

No complication occurred during insertion. No serious adverse events were reported during the 4-week healing period. Table 3 summarises procedural characteristics and the adverse events reported during the healing period.

**Table 3:** Results of the genital bar electrode insertion procedure and associated parameters

Phase	Variable	Number of patients or time Median (IQR)
Insertion	Successful insertion	14
	*Pain level* Very large extent Large extent Small extent Minimal or not at all	0 0 4 10
	*Bleeding during insertion* Mild bleeding* No bleeding	1 13
	Procedure duration**	25 min (22–30)
Healing	Healing time to Stage 3***	2 weeks (1–3)
	*Adverse events during healing* Pain Minor bleeding Swelling Redness Numbness Irritation Itching Adverse event reported by	7 7 4 3 1 1 1 10
	Genital bar electrode retained at 4-week evaluation	14

*Mild bleeding. Achieved quick haemostasis with compression

**This includes 15 min allocated to ensuring the proper functioning of anaesthesia

***Stage 1 (fresh/open wound), Stage 2 (wound is closed), or Stage 3 (skin channel has healed)

### Safety outcomes during 4-week stimulation period with genital bar electrode

**Fig. 3:**
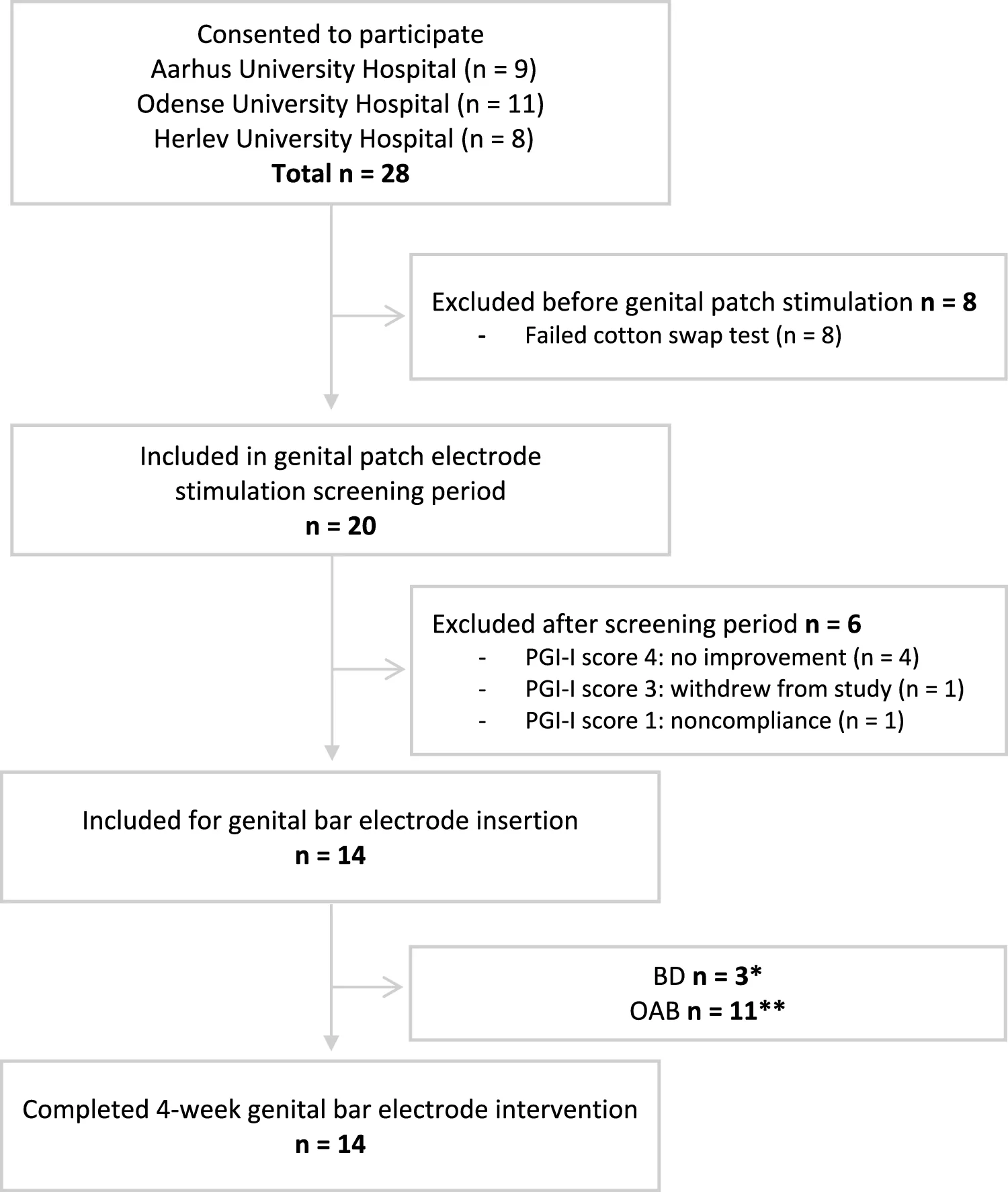
Flowchart of the patients in the study. *All patients with bowel dysfunction received 30 min of daily stimulation. **7 patients with overactive bladder received 30 min of daily stimulation; 4 patients with overactive bladder received on-demand stimulation

During the stimulation period, nine patients reported 14 adverse events, with a median of 1 (IQR 0–1). No serious or device-related adverse events occurred. The distribution of events included intense stimulation pain (*n* = 3), burning sensation in the labia (*n* = 2), urinary infection (*n* = 2), discomfort when wearing the device (*n* = 2), reduced bladder sensitivity (*n* = 1), thigh tingling during stimulation (*n* = 1), increased urinary urgency (*n* = 1), decreased sexual desire (*n* = 1), and granulation tissue formation (*n* = 1; Fig. 3).

### Performance outcomes during stimulation period with genital bar electrode and patch electrode

**Fig. 4:**
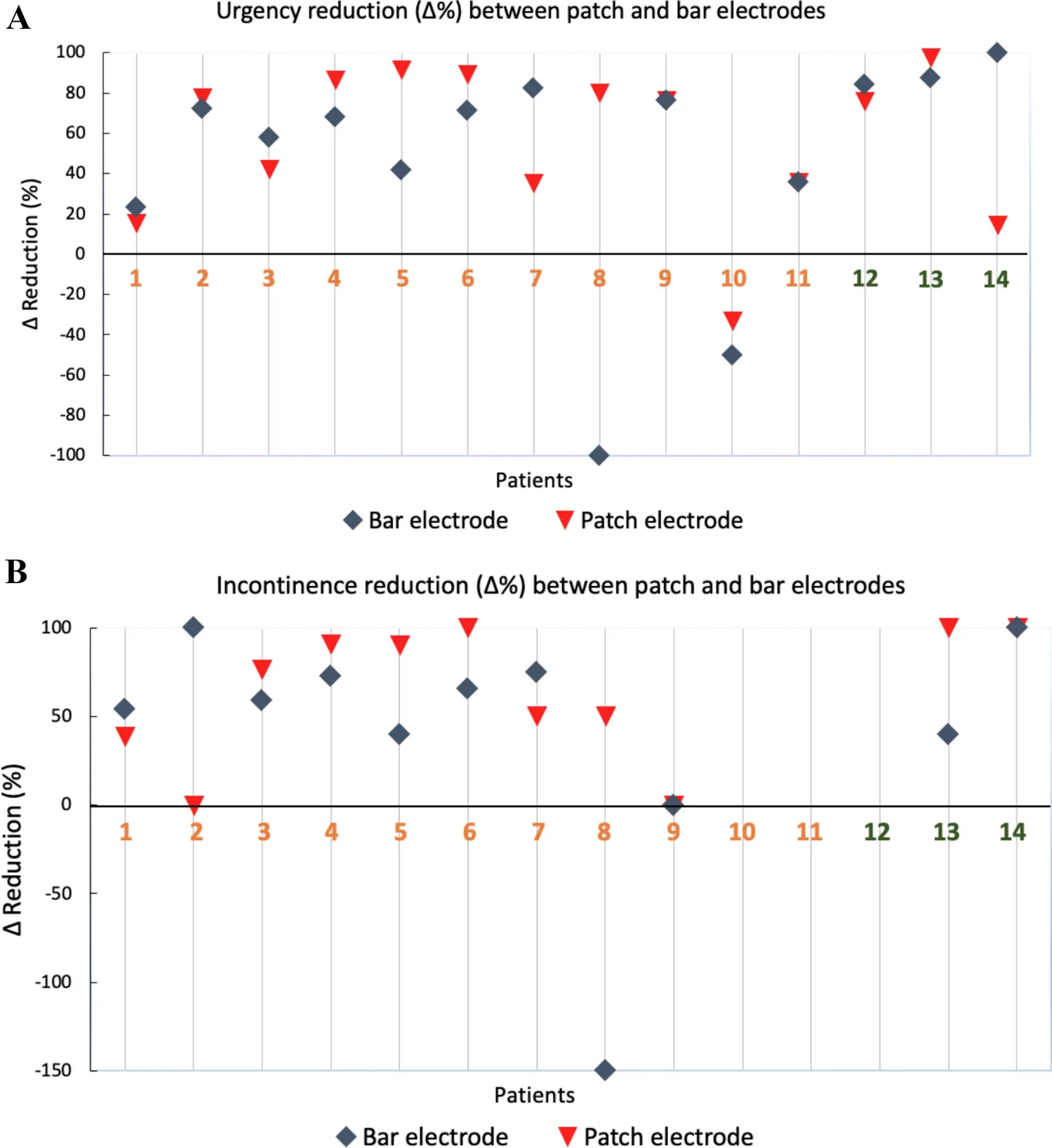
**A:** Individual reductions in urgency episodes after DGN stimulation with the bar and patch electrodes (*n* = 14). Patients 1–11 with OAB are marked in orange; patients 12–14 with BD are marked in green. **B:** Individual reductions in incontinence episodes after DGN stimulation with the bar and patch electrodes (*n* = 11). Patients 1–11 with OAB are marked in orange; patients 12–14 with BD are marked in green. Patients 10–12 did not experience incontinence at baseline

Median reduction in urgency episodes during the stimulation period with the genital bar electrode was 70% (IQR 36–82%) compared to baseline, non-significantly different from stimulation period with the patch electrode 76% (IQR 35–86%) (*P* = 0.489). Figure 4A shows the individual reductions in the number of urgency episodes for both electrode types (*n* = 14).

For patients with incontinence at baseline (*n* = 11), the median reduction in incontinence episodes during the stimulation period with the genital bar electrode was 59% (IQR 25–75%) compared to baseline, non-significantly different from stimulation period with the patch electrode 76% (IQR 38–100%) (*P* = 0.264). Figure 4B illustrates the individual reductions in the number of incontinence episodes for both electrode types (*n* = 11).

## Discussion

This feasibility study is the first to describe the insertion of a genital bar electrode as a novel approach for DGN stimulation in women with BD or OAB. The genital bar electrode may provide a more patient-friendly alternative to patch electrodes, which often detach or shift position during stimulation [27]. Our preliminary results indicate that the insertion procedure is feasible and safe. Furthermore, symptom improvements appear comparable with those observed using patch electrodes [27].

Insertion of the genital bar electrode is a minimally invasive procedure that was feasible and appeared safe under aseptic conditions with topical anaesthesia. It was well tolerated, with no serious perioperative complications, and was reported to be painless in 71% of patients. These findings align with the existing literature on genital piercings, in which complications are generally minor (bleeding, pain, swelling, or occasional infection), and serious adverse events are rare. Likewise, the adverse events observed in the present study were predominantly mild and self-limiting. However, the available evidence remains limited [33].

This study included only female patients, primarily for ethical reasons related to anatomical differences, as healing times for comparable piercings in male patients typically are several months. Future studies could adjust the protocol to include male patients, facilitating evaluation of the intervention in both genders.

During the 4-week healing period after genital bar electrode insertion, no serious adverse events were observed (Table 3). Adverse events were generally mild and transient. The most common were minor bleeding and mild pain; a single patient required paracetamol on two occasions. Swelling resolved with reduced physical activity. No infection or electrode migrations were reported. These observations offer valuable practical information concerning expected peri-procedural symptoms, patient counselling, and post-procedural management.

During the 4-week stimulation period with the genital bar electrode, no serious adverse or device-related adverse events occurred. Reported adverse events resolved in all cases except one instance of reduced sexual desire. Pain-related adverse events were not formally quantified; they were transient and managed by adjusting stimulation intensity, without requiring analgesic treatment. Granulation tissue around the electrode was observed in one patient and resolved after temporary cessation of stimulation and application of a silicone disc, allowing stimulation to resume without complications. Importantly, no patient chose to discontinue or remove the genital bar electrode as a result of adverse events.

Patient symptom diaries suggested reductions in symptoms in both BD and OAB for most patients. While responses in the patients with BD were more variable, the overall pattern indicates that genital bar electrode stimulation produces effects consistent with those observed using patch electrodes. However, the symptom-related findings should be interpreted cautiously and primarily support the physiological plausibility of effective stimulation using the genital bar electrode.

The open-label design may have introduced bias in patient-reported outcomes. The small sample size and short follow-up limit generalisability and preclude definitive conclusions regarding efficacy or long-term safety. The study was not designed or powered to evaluate clinical effectiveness, and the symptom-related findings should be considered exploratory. Furthermore, the heterogeneous study population may limit the interpretation of symptom-related outcomes. Two patients did not want to progress from the screening phase with the patch electrode to insertion of the genital bar electrode, despite reporting subjective improvement. This indicates that the intervention may not be acceptable or suitable for all patients, with cosmetic and personal considerations influencing treatment uptake in addition to perceived efficacy. The three-centre design enhances applicability but may introduce procedural variability, although training sessions were completed together. Despite these limitations, preliminary symptom response outcomes provide early insights into clinical benefit.

While insertion appears feasible and safe, trained personnel are required. Before broader clinical adoption, standardised training, clear procedural guidelines, and defined patient selection criteria are needed. Future studies should first evaluate longer-term safety and durability of genital bar electrode placement, including potential late infections, electrode removal and effects on sexual function, followed by controlled studies assessing comparative efficacy against established neuromodulator therapies.

## Conclusion

The insertion of the genital bar electrode was feasible and appeared safe in this short-term study. Preliminary findings suggest reduced episodes of urgency and improved continence with the genital bar electrode, with symptom improvements similar to those reported with the patch electrode. Larger studies with longer follow-up are needed to further evaluate safety, acceptability, and clinical effectiveness.

## Supplementary Information

Below is the link to the electronic supplementary material.

Supplementary file1 (DOCX 309 KB)

## Data Availability

Data are available from the authors upon reasonable request.
