# Supplementary material for: Genital bar electrode for dorsal genital nerve stimulation: feasibility and safety of a novel approach for managing bowel dysfunction and overactive bladder
Source: Tech Coloproctol. 2026 Jul 9;30(1):150. doi: 10.1007/s10151-026-03386-6 (PMC13630791; doi:10.1007/s10151-026-03386-6)
Supplement: Supplementary file 1 [file 10151_2026_3386_MOESM1_ESM.docx]

Appendix A: Patient Care Instructions.
